# A simple and cost-effective detection of capsule origin source using FTIR-ATR

**DOI:** 10.1007/s13197-025-06361-5

**Published:** 2025-06-18

**Authors:** Radhiahtul Raehan Mustafa, Mohammad Naqib Hamdan, Siti Nur Hadis A. Rahman, Ida Madiha Yusoff, Nor Suhada Amin, Qosrina Abdullah

**Affiliations:** 1 Faculty of Social Sciences and Humanities, Academy of Islamic Civilisation, Universiti Teknologi Malaysia, UTM Skudai, 81310 Johor, Johor Bahru Malaysia; 2 Institute of Bioproduct Development, Universiti Teknologi Malaysia, UTM Skudai, 81310 Johor, Johor Bahru Malaysia; 3 Faculty of Management, Universiti Teknologi Malaysia, UTM Skudai, 81310 Johor, Johor Bahru Malaysia

**Keywords:** Discriminant analysis, FTIR-ATR, Porcine, Non-porcine, Gelatin, Halal detection

## Abstract

The adulteration of food by undeclared ingredients is a major problem for both consumers and regulatory authorities. The increasing demand for halal pharmaceuticals emphasises the need for a robust technique to identify porcine-derived components in samples. Fourier transform infrared spectroscopy coupled with attenuated total reflectance (FTIR-ATR) has emerged as a rapid, non-destructive and cost-effective technique for the detection of porcelain-derived components. In this study, 13 gelatine capsule samples from different sources were analysed with FTIR-ATR to investigate the presence of porcine and non-porcine. The application of discriminant analysis to the FTIR-ATR data allowed successful differentiation between porcine and non-porcine components in the samples. The infrared spectra revealed prominent functional groups, such as the amino group and the carbonyl group, which are characteristic of gelatin. While the spectra of the samples showed striking similarities due to their common major component, subtle differences were observed in the specific ranges of 3600–3000 cm^−1^ and 1700–1000 cm^−1^. This integrated approach of FTIR-ATR and discriminant analysis proved to be a robust and reliable method for authenticating capsule origin and a useful tool for tracing halal detection in capsule products.

## Introduction

Pharmaceutical products have emerged as one of the promising sectors in the global halal market (Mohtar et al. 2014). Among the key ingredients of concern is gelatin, widely use in pharmaceutical capsules but raises significant religious concerns for Muslim consumers due to its potential porcine origin (A-Jalil 2018). Gelatin is a solid, translucent and nearly tasteless substance derived primarily from collagen, a fibrous protein extracted from animal sources such as bovine hides, bones, porcine skin and marine organisms (Said 2020). For instance, while bovine gelatin is typically sourced from cattle bones and hides, porcine gelatin is predominantly obtained from pigskins, which raise significant halal compliance issues.

**Fig. 1:**
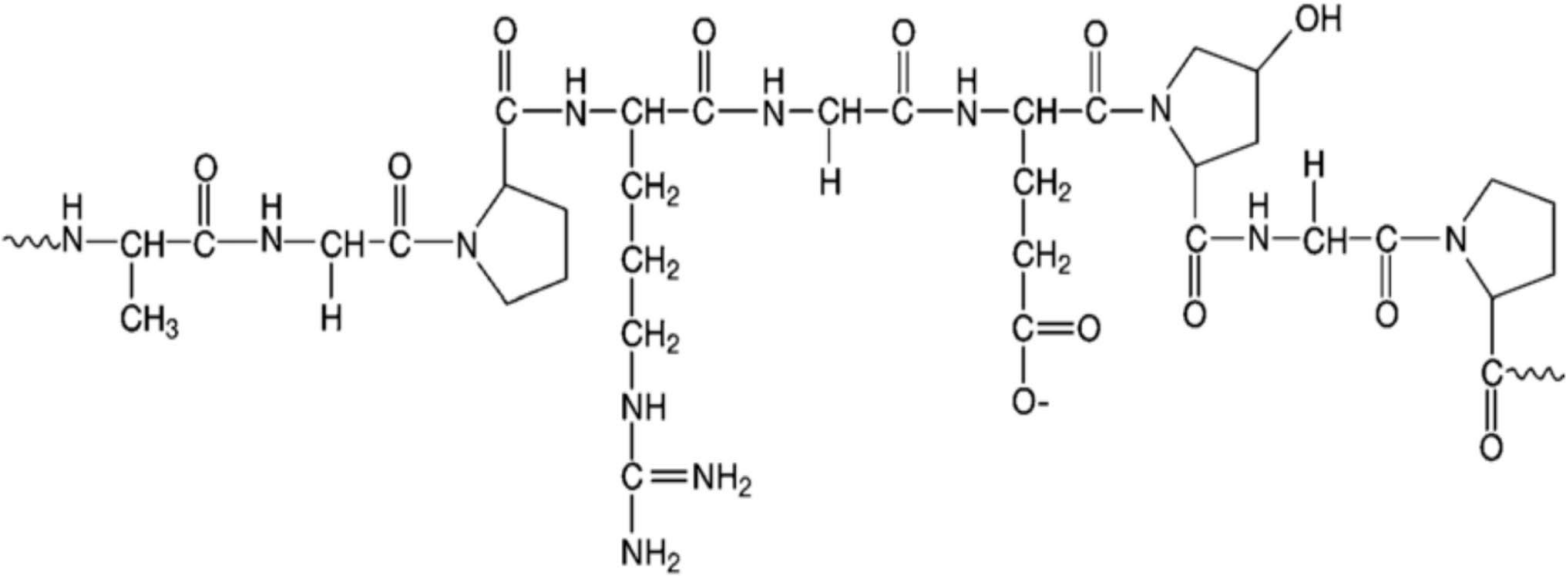
Chemical structure of gelatin (Said 2020)

Structurally, Gelatin (Fig. 1) is a heterogeneous mixture of single or multi-stranded polypeptides formed through the partial hydrolysis of collagen (Said 2020). As the main protein in animal bones, skin, and connective tissues, it consists of 300–4000 amino acids, arranged in left-handed proline helix conformations (Farris et al. 2010). A single gelatin molecule has an average molecular mass of 95 kDa, with dimensions of 1.5 nm in width and 0.3 mm in length. Due to its versatile functional properties, gelatin is extensively used across industries, from food (e.g., dairy products, desserts, gummy candies) to photography, and pharmaceuticals. Table 1 further highlights the prevalence of pig-derived ingredients in everyday products, underscoring the need for halal alternatives.

**Table 1:** Pig-derived ingredients in daily products (Ishaq et al. 2020)

Organ	Products
Bile, Pancreas, Intestines and other organ	Food supplement, Insulin, Sausage casing, coagulant (heparin) Fermentation medium, chemical weapons testing, tamborine (bladder)
Meat	Processed food, food product such as ham, sausage, and burger patties
Fat and oil	Cosmetics, flavorings and colorant, shortening, medicine tablets, butter sausage additive, cake, bread, biscuit, fabric softener, washing powder, shampoo, soap toothpaste, candle, pain and crayon, cooking oil additive, snack, cosmetic, flavorings, noodles
Blood/serum Bone, Gelatin, Emulsifier and Food stabilizer Skin, Accessories and Collagen Hair	Sausage, vaccine, tablets, fermentation medium, colorant (dog treat), and photodynamic therapy Active carbon, calcium, phosphorus (toothpaste, milk, bone ash: train brakes), Paper (improve stiffness and reduce moisture), gun powder/cordite (transport to bullet casing, cork (binder), photographic film Yoghurt, ice cream, butter Fruit juices, margarine, beverages, cream cheese. Leather bag, wallet, shoes, jacket, Cosmetic, sausage casing, hide and Glue for wood working industry, Brushes (paints brush, pantry brush, toothbrush), high protein flour, fur coat, fertilizer and dough softener

Porcine, or pig-derived, materials are commonly utilized as a source of gelatin due to their cost-effectiveness, availability, and high breeding rates (Manan et al. 2017). This practice, however, poses a significant challenge for Muslim consumers, as gelatin derived from porcine sources is considered haram (forbidden) according to Islamic principles (Kurnia et al. 2023). The Quran explicitly prohibits the consumption of pork in verse 173 of Chapter Baqarah, and in verse 115 of Chapter Nahl, it is further stated that “He has only forbidden to you dead animals, blood, the flesh of swine, and that which has been dedicated to other than Allah” (Andreani et al. 2023).

Given these religious restrictions, Muslim consumers actively seek assurances that the gelatin used in pharmaceutical products, including capsules, is sourced from halal (permissible) animals (Mardian et al. 2021). This concern extends beyond just the raw materials, as the entire supply chain, including transportation, distribution, and storage, must also adhere to halal principles. The complexity of modern production and distribution systems makes it increasingly difficult for consumers to ensure the halal status of a product, underscoring the need for comprehensive halal certification and traceability throughout the supply chain.

The Malaysian halal standard, which was introduced in 2003, comprises three main elements: Malaysian Halal Standard MS 1500:2004 (Halal Foods–Production, Preparation, Handling and Storage–General Guidelines), Malaysian Halal Standard MS 1900:2005 (Quality Management System–Islamic Perspectives) and Malaysian Halal Standard MS 22200-1:2008 (Islamic Consumer Products–Part 1: Cosmetics and Personal CareProducts–General Guidelines). The introduction of the Malaysian halal standard is in line with Malaysia’s vision to become a global halal centre and set a benchmark for international halal standards (Alipal et al. 2021). The concept of halal authentication emphasises the importance of healthy and high-quality products that include acceptable ingredients, hygienic practises throughout production, handling and processing, hygienic transportation and slaughtering of meat according to Islamic rites (Djagny et al. 2010).

Obtaining halal certification for pharmaceutical items, including gelatin capsules, requires establishing that the sources are devoid of non-halal compounds and that animals are slaughtered in accordance with Sharia law (Djagny et al. 2010). The MS 2636 standard classifies animals as either land or aquatic. All land animals are considered halal, except for those explicitly forbidden in the Quran, those not slaughtered according to Shariah law, predators with tusks, and toxic creatures. Similarly, all aquatic animals are deemed halal, with the exception of those that are dangerous or toxic, amphibians, and those prohibited by Shariah law and fatwas.

Furthermore, Halal certification requires that ingredients be naturally sourced and free from harmful substances. All plant varieties and derivatives are inherently deemed halal unless poisonous or hazardous to human health. However, gelatin presents a critical exception to this principle, as its various animal-derived sources which are widely used in food and pharmaceutical manufacturing which introduce significant halal compliance challenges. Unlike plant-based materials, gelatin’s permissibility depends entirely on traceable, non-porcine origins and ethically sound processing methods, necessitating rigorous verification protocols.

The pharmaceutical industry has developed several plant-based and halal-certified alternatives that maintain similar functional properties. Plant-derived hydrocolloids such as pectin (extracted from citrus or apple peels) and carrageenan (obtained from seaweed) have proven effective as gelling agents in capsule production and film coatings. It offers comparable textural properties to traditional gelatin (Mahamud et al. 2023). Similarly, agar–agar, a polysaccharide derived from red algae, has been successfully utilized in both hard and soft capsule formulations due to its thermo-reversible gelling characteristics (Waraczewski et al. 2022).

For a more innovative approach, microbial fermentation techniques using *Escherichia coli* or yeast have enabled the production of recombinant gelatin that is both animal-free and halal-compliant, with consistent quality and purity (Gómez-Guillén et al. 2011). Moreover, Fish gelatin source from the skins and scales of halal-certified fish species, presents another viable alternative, although its gel strength and melting points may vary depending on the fish source (Karim and Bhat 2009). Additionally, synthetic polymers like hypromellose (HPMC) and pullulan have gained widespread acceptance as cellulose-based substitutes for capsule shells, providing excellent film-forming properties and compatibility with pharmaceutical ingredients (Dobariya et al. 2021).

Despite offering halal-compliant solutions, plant-based gelatin alternatives face significant challenges, including higher production costs (20–30% more expensive than animal gelatin) due to complex extraction processes (Waraczewski et al. 2022), weaker gel strength and thermal instability, requiring additives for comparable functionality (Ghosh et al. 2022). Additionally, batch-to-batch variability in plant sources complicates standardization (Mazuki and Bhari 2024), while seasonal raw material fluctuations affect consistency (Karim and Bhat 2009). Regulatory hurdles, such as lengthy approvals and inconsistent halal certification standards, further limit widespread adoption (Gómez-Guillén et al. 2011). These factors hinder their viability as direct replacements for conventional gelatin in pharmaceuticals.

Consequently, animal-derived gelatin remains the most functionally effective material for pharmaceutical and food applications. However, a significant challenge arises from the difficulty of tracing its biological origin in processed products, which increases risks of adulteration and mislabeling. This ambiguity in sourcing is particularly problematic for halal-certified products like capsules, where origin verification is critical. Thus, robust analytical methods are urgently required to accurately identify gelatin sources and ensure compliance with halal standards.

Analytical methods for the detection of non-halal ingredients, including pork-derived capsules, such as gas chromatography–mass spectrometry (GCMS), liquid chromatography–mass spectrometry (LCMS), Western blot, polymerase chain reaction (PCR) and enzyme-linked immunosorbent assay (ELISA) are notable for specific qualitative and quantitative detection (Rohman et al. 2020). However, these methods are costly, require technical expertise, tedious sample preparation and are time consuming.

For many decades, Fourier transform infrared spectroscopy (FTIR-ATR) has played a crucial role in halal analysis. With this technique, porcelain-derived materials can be distinguished from halal sources such as cattle, even if they have similar properties. FTIR-ATR offers significant advantages, including simplicity and cost-effectiveness, which are suitable for the detection of samples. Therefore, FTIR-ATR was used in this study to identify the presence of porcine-derived ingredients in the capsules.

## Material and methods

### Sample

A total of 13 capsule gelatin samples were obtained from different sources. Porcine and bovine gelatine powder were used as a control in this study.

### Sample preparation

The capsule gelatin was dissolved and homogenized in deionized water until a clear solution was obtained. Meanwhile, the powder sample was prepared for pellet formation using the KBr method prior to infrared spectroscopy measurements. First, a small amount of gelatin powder was finely ground and mixed with a large excess of infrared-grade KBr. The homogenous mixture was then compressed into a translucent pellet (diameter: 13 mm) using a hydraulic press 15 tons pressure. The pellet was then analyzed using FTIR spectrometer to obtain the absorbance spectrum for characterization.

### Infrared spectroscopy measurements

FTIR (Perkin Elmer, Waltham, USA) was employed as a qualitative method to determine the functional groups of gelatine. Samples were placed in direct contact with a horizontal ATR, set up using a ZnSe crystal with an aperture angle of 45 °C and a refractive index of 2.4 at 1000 cm^−1^, at a controlled ambient temperature (Smart Ark, with dimensions of 1.0 × 6.0 cm). FTIR was equipped with a deuterated triglycine sulphate (DTGS) detector and connected to the OMNIC software operating system (Version 7.0, Thermo Nicolet) for processing and evaluating infrared spectra. Functional groups of gelatine in the chemical structure were identified at the range of 4000–650 mm by co-adding 32 scans at a resolution of 4 cm^−1^ wavelength. The ATR crystal was carefully cleaned with acetone before being placed on the next sample. A new reference air background spectrum was taken after every scan.

## Results and discussion

The accurate determination of the source of gelatin in capsule is of paramount importance for both Islamic regulatory compliance and consumer awareness. FTIR spectroscopy has emerged as a promising analytical method for this purpose, which offering advantages such as being a non-destructive technique, requiring minimal sample preparation, less sample, and provide rapid analysis for determining halal status of the product (Cebi et al. 2019).

**Fig. 2:**
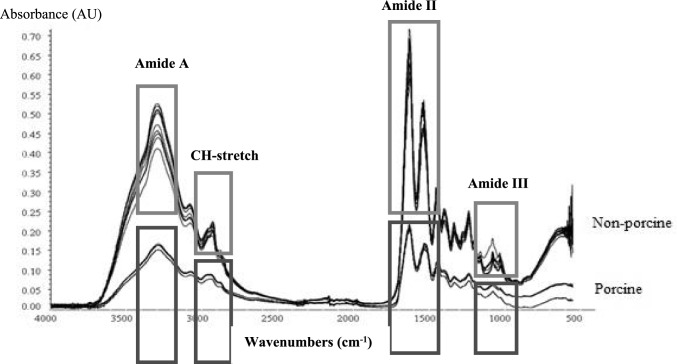
FTIR-ATR spectra of porcine (red) and non-porcine (blue) gelatin samples. Key vibrational bands are labeled: Amide I (1650 cm⁻^1^, C=O stretching), Amide II (1550 cm⁻^1^, N–H bending/C–N stretching), and CH-stretching (2930 cm⁻^1^, aliphatic C-H vibrations)

Molecular structure of gelatin is formed by several functional groups, constituting the main protein structure. It is bonded by a combination of amino acids (N–H), which are joined together by amide bonds. Functional groups were identified through absorption bands and undergo vibration releasing energy upon light absorption (Dong et al. 2017). It identifies non-porcine samples by detecting molecular vibrations and analyzing the interaction between infrared light and chemical bonds (Alkhuder 2022). Infrared spectra explore a diverse range of bonds and functional groups, including hydroxyl (O–H), amine (N–H), and carbonyl (C=O). Consequently, the resulting infrared spectrum demonstrates peaks representing the distinctive bonding characteristics within the compounds (Tabil et al. 2011). Figure 2 illustrates the results of FTIR spectra, distinguishing between porcine and non-porcine gelatin.

In Fig. 2, the FTIR spectra of non-porcine gelatin are depicted to be higher than those of porcine gelatin. The major peaks observed in the mid-infrared region, ranging from 3600 to 3000 cm^−1^ and 1700 to 1000 cm^−1^. The FTIR spectra of the samples contain the region of Amide A, Amide I, Amide II and Amide III. These regions were divided according to the stretching of NH, CH_2_ and CO (Sadat and Joye 2020). The result obtained represented similar spectral patterns of regions with previous study at Amide A (3000–2500 cm^−1^), Amide I (1600–1500 cm^−1^), Amide II (1560–1335 cm^−1^), and Amide III (1240–670 cm^−1^) (Irfanita et al. 2022). From the result, the spectra showed similar spectra among the samples due to the similarity of the main component composed in capsule samples which is gelatin.

FTIR-ATR analysis effectively differentiated porcine and non-porcine gelatin based on characteristic amide band patterns. Additional instrumental methods, including LC–MS and GC–MS, were employed for gelatin identification. LCMS has proven effective in detecting species-specific biomarkers such as porcine collagen peptide which were detected at m/z 1135 (Zhang et al. 2022), while GCMS can identify sterol profiles that distinguish mammalian gelatins (Darmawan et al. 2024). The higher Amide A peaks observed in the FTIR spectra for non-porcine samples may correlate with bovine-specific peptides detectable by LCMS, and the absence of cholesterol markers in GCMS could further verify plant/fish origins where FTIR shows ambiguous Amide III patterns. Table 2 presents the vibrational frequencies associated with the functional groups identified in the IR spectra of gelatin.

**Table 2:** Functional groups vibration of gelatine

Region	Peak wavenumber cm^−1^													Assign
	NH1	NH2	NH3	HS1	HS2	HS3	HS4	HS5	HS6	HS7	HS8	HS9	HS10	
Amide A	3287	3290	3284	3302	3296	3296	3287	3293	3293	3296	3296	3293	3293	N–H stretch couple with Hydrogen bonding
	3075	3061	3052	3075	3072	3081	3078	3069	3072	3075	3075	3078	3069	N–H stretch couple with Hydrogen bonding
	2919	2949	2937	2925	2925	2928	2925	2922	2925	2931	2931	2925	2943	CH_2_ asymmetrical stretch
Amide I	1627	1633	1633	1630	1533	1633	1627	1630	1633	1627	1630	1630	1627	C-O stretch, Hydrogen bonding coupled with -COO
	1524	1527	1533	1536	1533	1539	1539	1536	1539	1542	1542	1539	1539	NH bend coupled with CN stretch
	1444	1442	1447	1450	1450	1450	1450	1450	1444	1450	1450	1450	1453	CH_2_ band
	1333	1333	1333	1336	1394	1339	1330	1400	1336	1336	1400	1394	1336	CH_2_ wagging of proline
Amide III	1235	1233	1235	1235	1235	1235	1235	1536	1235	1233	1235	1235	1235	N–H bend
	–	–	–	1168	1026	1159	–	1029	1029	1153	1168	1179	1162	C-O stretch
	–	–	1079	1079	1077	1079	1079	1076	1079	1079	1079	1079	1082	Skeletal stretch

The differences in vibration modes manifest within the peptide bond, with major functional groups such as the amine group (N–H), amide (N–C=O), and carbonyl group (C=O) being evident in the results of FTIR spectroscopy. Similar spectra peaks and patterns was obtained by Cebi et al. (2019), where gelatin was analyzed in the mid-infrared region at 4000–500 cm^−1^ correspond to the stretching of NH, CH_2_, and CO. The functional group vibration of Amide A contains N–H stretch couple with hydrogen bonding and CH_2_ asymmetrical stretch. Meanwhile for Amide I, the peak assigns the C–O stretch, hydrogen bonding coupled with carboxylic acid (–COO), NH bend coupled with CN stretch, CH_2_ band and CH_2_ wagging of proline. The functional group vibration of Amide III represents N–H bend, C–O stretch and skeletal stretch (Cebi et al. 2019).

The combination of FTIR with Partial Least Square (PLS) analysis enables fast and simultaneous qualitative analysis of complex mixtures. The IR spectra of the samples were analysed using chemometric methods. In particular, the classification and characterisation of the samples was carried out using principal component analysis (PCA). PCA was performed using TQ Analyst software to identify spectral features that significantly affect the spectral variance. Sohar (2012) reported the use of a similar calibration model focusing on the FTIR spectral ranges of 3290–3280 cm^−1^ and 1660–1200 cm^−1^ to determine the origin of unknown gelatin samples.

Discriminant analysis, combined with FTIR and analytical software, is proving valuable for building classification models. This approach uses techniques such as Mahalanobis distance, which calculates the distance between unknown samples and known sample classes, to aid in identification and categorisation (Fahelelbom et al. 2022). This method has found application in various fields, including pharmaceutical analysis and disease diagnostics, demonstrating its versatility and effectiveness in classification tasks (Naseer et al. 2020).

The FTIR spectra of the samples were divided into two groups for discriminant analysis: a calibration group and a validation group. The calibration spectra were used to create a discriminant model, while the validation spectra were used to check the accuracy of the model. This standard practice of dividing the data into calibration and validation groups is crucial for creating robust and reliable models (Jiao et al. 2020). The validation and calibration groups included spectra from porcine and non-porcine samples, as listed in Table 3.

**Table 3:** Group of calibration and validation spectra

Index	Sample	Analysis
1	NH-1	Validation
2	NH-2	Calibration
3	NH-3	Calibration
4	HS-1	Validation
5	HS-2	Calibration
6	HS-3	Calibration
7	HS-4	Calibration
8	HS-5	Calibration
9	HS-6	Calibration
10	HS-7	Validation
11	HS-8	Calibration
12	HS-9	Calibration
13	HS-10	Calibration

**Fig. 3:**
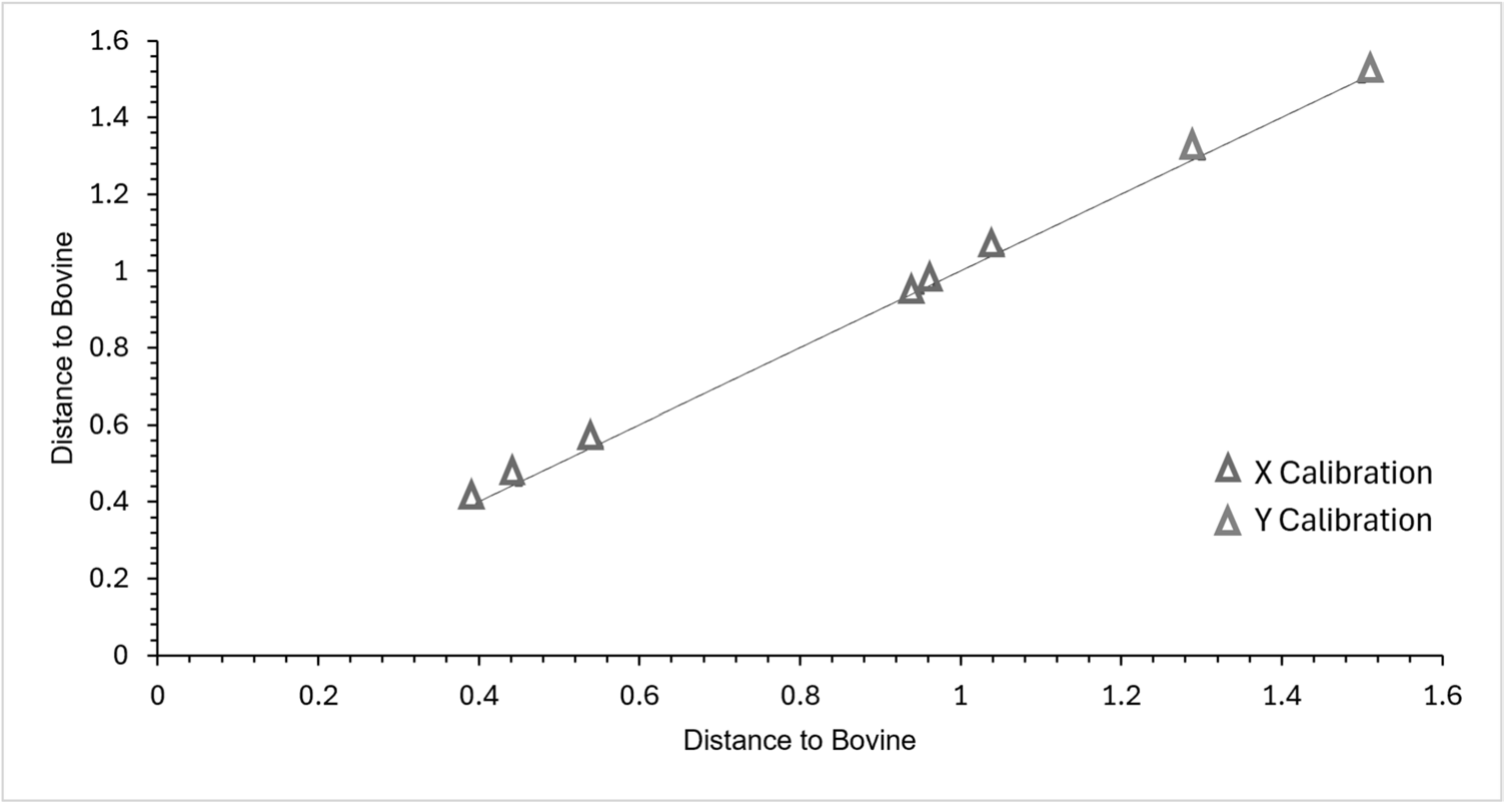
Clustering analysis of non-porcine samples using TQ analyst (Distance to Bovine vs. Calibration Axes)

Calibration spectra are used to create a discriminant model, while a separate group of spectra is used for validation to check the model. The validation and calibration groups of the statistical models were derived from porcine and non-porcine samples. Up to three IR spectra from each class were used for validation, while most of the FTIR spectra were used as calibration spectra in each class. A discriminant model was created with the set of calibration spectra and then verified with the set of validation spectra. The validation and calibration spectrum groups were selected using TQ Analyst software. A calibration sample was evaluated to determine the quality of the samples. Meanwhile, the validation sample was used to verify the calibration model. Figure 3 shows a Mahalanobis distance plot for the non-porcine source in the samples.

As shown in Fig. 3, all the validation spectra were within the sample groups. Therefore, the model in this study was considered correct since all the validation spectra fell within their respective groups. A Mahalanobis model was obtained with the samples clearly separated and identified between the non-porcine and porcine sources. The samples were separated according to their characteristics. The ten samples of non-porcine origin were aligned in one group, proving that all ten samples were non-porcine, while the remaining samples were of porcine origin. Three spectra were selected for validation, and the others were used for calibration. The analysis began with the classification process, which was based on the characteristics of the spectra, such as peak height, location, area, width, ratio, and noise, with spectra from different sources and known categories collected.

## Conclusion

The study demonstrates the utility of ATR-FTIR spectroscopy in conjunction with discriminant analysis as a rapid, non-destructive and sensitive technique for the detection of porcine and non-porcine sources in capsules. The combination of ATR-FTIR and chemometric techniques, such as discriminant analysis, has been shown to be effective in distinguishing between different classes of samples and identifying specific chemical signatures. This approach has been successfully applied to discriminate between porcine and non-porcine capsules, with key differences observed in the wavelength of 3600–3000 cm^−1^ and 1700–1000 cm^−1^. The results indicate that although the overall spectra of the two sources were quite similar, subtle differences in peak intensities within specific regions could be detected using the proposed analytical approach. Fourier transform infrared spectroscopy has proven to be a powerful tool for the authentication and detection of adulteration in various gelatin-containing food and pharmaceutical products.

## Data Availability

The authors declare that the data supporting the findings of this study are available within the manuscript.

## Code availability

Non-applicable.
